# Cysteinyl leukotriene correlated with 8-isoprostane levels as predictive biomarkers for sensory dysfunction in autism

**DOI:** 10.1186/s12944-016-0298-0

**Published:** 2016-08-17

**Authors:** Hanan Qasem, Laila Al-Ayadhi, Afaf El-Ansary

**Affiliations:** 1 Central Laboratory, Female Center for Medical Studies and Scientific Section, King Saud University, Riyadh, KSA Saudi Arabia; 2 Biochemistry Department, Science College, King Saud University, P.O. Box 22452, Riyadh, 11495 Saudi Arabia; 3 Autism Research and Treatment Center, Riyadh, Saudi Arabia; 4 Shaik AL-Amodi Autism Research Chair, King Saud University, Riyadh, Saudi Arabia; 5 Department of Physiology, Faculty of Medicine, King Saud University, Riyadh, Saudi Arabia; 6 Medicinal Chemistry Department, National Research Centre, Dokki, Cairo Egypt

**Keywords:** Autism, Oxidative stress, Neuro-inflammation, 8-isoprostane, Cysteinyl leukotriene, Childhood autism rating scale, Social responsiveness scale, Short sensory profile

## Abstract

**Background:**

Autism is a neurodevelopmental disorder that clinically presented as cognitive deficits, social impairments and sensory dysfunction. An increasing body of evidence has shown that oxidative stress and inflammation are involved in the pathophysiology of autism. Recording biomarkers as measure of the severity of autistic features might help in understanding the pathophysiology of autism.

**Methods:**

This study investigates the plasma levels of 8-isoprostane and Cysteinyl leukotrienes (CysLTs) in 44 autistic children and 40 healthy controls. The recruited autistic patients were assessed for behavior, cognitive and sensory deficits by using different autism severity rating scales, including the Childhood Autism Rating Scales (CARS), Social responsiveness scale (SRS) and Short Sensory Profile (SSP). Receiver Operating Characteristics analysis (ROC) of the obtained data was performed to measure the predictive value of 8-isoprostane and Cysteinyl leukotrienes (CysLTs) as oxidative stress- related parameters. Pearson’s correlations between the measured parameters was also performed.

**Results:**

The concentrations of 8-isoprostane and CysLTs in autistic patients were significantly higher than those in controls. While cognitive and social impairments did not show any significant differences, the SSP results were strongly correlated with the levels of both of the biomarkers assessed. However, autistic children showed improvements in oxidative stress status (as determined by 8-isoprostane levels) at increasing ages.

**Conclusion:**

This study indicates that 8-isoprostane and CysLTs can be used as markers for the early recognition of autistic patients through sensory deficits phenotypes which might help early intervention.

## Background

Autism is a neurodevelopmental disorder that is characterized by social interaction impairment, repetitive behavior, cognition difficulties, and sensory dysfunction [1–3]. Approximately 1 out of 50 children are currently affected with autism in the US [4]. As multifactorial disorder, autism is considered a highly heterogeneous disorder with many causes and associated factors resulting in variable ranges of clinical presentations and severity. Genetics, environmental interaction, impaired detoxification, immune dysregulation, inflammation and oxidative stress are the main factors contributed in autism pathogenesis; a clear cause for autism has not been identified to date [5, 6].

Neuro-inflammation, which is described as specific and chronic glial reactions that occur in the CNS, has been implicated in autism [7, 8]. It can cause brain damage through increased proinflammatory cytokine release and abnormal neuronal growth [8–10]. Oxidative stress is usually thought to be the major contributor and the primary cause of brain damage and inflammation [11, 12].

In the early developmental stages of childhood, the vulnerability of the brain to oxidative stress, measured as abundance of reactive oxygen species (ROS), low antioxidant capacity, and high levels of lipid content is identified as a major factor that might increase oxidative stress in autism [13]. Many studies have reported that lipid peroxidation products are much higher in autistic patients compared to healthy controls [14–16]. In addition, decreased levels of antioxidants and detoxifying agents in samples from autistic children has been reported [17–20]. A strong correlation has been observed between low levels of the antioxidants and impairments in language skills [14].

Polyunsaturated fatty acid residues in membrane phospholipids are very sensitive to oxidative stress; ROS may induce the breakdown of lipids, which can disrupt the composition of membrane phospholipids and alter neuronal function. Proinflammatory cytokines that are released from glial cells can up regulate the transcription of arachidonic acid (AA) metabolism-related genes, among which are cytosolic phospholipase A2 (cPLA2), secretory PLA2 (sPLA2), and cyclooxygenase-2 (COX-2) [21–23].

The released AA can be converted through the action of COX and lipoxygenase (LOX), into the proinflammatory lipid mediators, prostaglandins (PG) and leukotrienes, respectively or through non-enzymatic free radical oxidation to isoprostanes by [24, 25].

8-isoprostane, which is the most sensitive indicator of oxidative stress and a perfect marker of redox dysfunction, has been discovered to play an important role in some neurological disorders [26–28]. Cysteinyl leukotrienes (CysLTs) as biologically active lipid mediators play a key role in mediating inflammation and involved in pathological states with an inflammatory components. They enhance the activity of inflammatory cells such as monocytes, eosinophils, mast cells and T lymphocytes [29]. Increased levels of 8-isoprostane, CysLTs, and impaired levels of phospholipids and fatty acids have been recorded in children with autism compared to controls [30–33].

Social impairments and cognitive deficits are core features of autism. In autistic patients, difficulties in understanding the facial expressions of others, attention deficits, and communication problems are very common symptoms [34–40]. Moreover, children with autism have abnormal auditory, visual, tactile and oral sensory processing that is significantly different from healthy controls [41–43].

Few studies have drawn correlations between changes in oxidative stress biomarkers and autism severity. CARS, SRS and SSP are autism severity scales that have been designed to measure cognitive deficits, social impairments and sensory dysfunction, respectively. This study aims to determine the biochemical correlation between 8-isoprostane, CysLTs, age, and autism severity scales in an attempt to clarify the role of oxidative stress (which is indicated by 8-isoprostane levels) and inflammation (which is indicated by CysLTs levels) in the etiopathology of autism.

## Methods

### Materials

#### Chemicals

The hexane, ethyl acetate, hydrochloric acid and methanol that were used for extraction of cysteinyl leukotrienes for analytical experiments were obtained from, Winlab, Merck, Kock-light laboratory England and BDH, respectively.

#### Participants

Forty-four children with autism were recruited from the Autism Research and Treatment Center-King Khalid University Hospital, and 40 healthy controls were recruited from the Pediatric Laboratory Center of King Saud Medical City in Riyadh. Parental consent for these studies was obtained as approved by the ethical guidelines of medicine of King Saud University according to the most recent Declaration of Helsinki (Edinburgh, 2000). A diagnosis of autism was confirmed in all subjects using the Autism Diagnostic Interview-Revised (ADI-R), the Autism Diagnostic Observation Schedule (ADOS) and the Developmental, Dimensional and Diagnostic Interview (3DI). The mean age of all autistic children participating in the study was 7 ± 4 years old, and all were simple cases. All participants were negative for fragile x gene mutations. The control group was recruited from the pediatric clinic of King Saud Medical City in Riyadh and had a mean age of 7 ± 3 years old. Subjects were excluded from the investigation if they had (e.g., seizures), psychiatric (e.g., bipolar disorder) or medical conditions. All participants were screened via parental interview for current and past physical illness. Children with known endocrine, cardiovascular, pulmonary, liver, kidney or other medical conditions were excluded from the study.

### Methods

#### Behavioral assessment

The CARS score was fulfilled as a scale for autism severity. CARS assesses the child on a scale from one to four in each of 15 dimensions or symptoms (including the ability to relate to people; emotional response; imitation; body use; object use; listening response; fear or nervousness; verbal communication; non-verbal communication; activity level; level and reliability of intellectual response; adaptation to change; visual response; taste, smell and touch response; and general impressions). A total score of at least 30 strongly suggests the presence of autism. Children who score between 30 and 36 have mild to moderate autism while those with scores between 37 and 60 points have severe autism [44]. SRS is the first widely used quantitative parent/teacher-report measure of autistic behaviors and is completed in 15 to 20 min. A total SRS score of 76 or higher is considered severe and is strongly associated with a clinical diagnosis of autistic disorder. A score between 60 and 75 is in the mild to moderate range of social impairment [45].

The Short Sensory Profile [46] is a 38-item questionnaire intended to rate a variety of sensory impairments. Each item on the SSP is measured on a 5-point Likert scale. Domain scores are measured in the areas of tactile sensitivity, taste/smell sensitivity, movement sensitivity, seeking sensation, auditory filtering, low energy levels, and visual/auditory sensitivity. Domain scores and the overall sensory response are categorized as typical performance, probable difference from typical performance, or definite difference from typical performance. Scores less than 142 indicate severe performance (definite difference from typical performance), scores between 142 and 152 indicate mild to moderate performance (probable difference from typical performance) and scores between 153 and 190 indicate typical performance. The SSP can provide information about the sensory processing skills of children with autism to assist occupational therapists in assessing and planning interventions for these children [41].

#### Blood sample collection

After overnight fasting, blood samples from 44 autistic children and 40 healthy controls were drawn from the arm by a qualified lab technician. Blood was taken into 3-ml blood collection tubes containing EDTA, and samples were immediately centrifuged at 4 °C at 3000 g for 20 min. The plasma was decanted, dispensed into four 0.75 ml aliquots (to avoid multiple freeze-thaws cycles) and stored at −80 °C until analysis.

#### Biochemical assays

The concentrations of 8-isoprostane and CysLTs were measured in plasma of control and autistic subjects. All biochemical analyses were performed in duplicate, and the mean values were reported.

#### Sample extraction for cysteinyl leukotrienes

300 μl of plasma samples were acidified with 30 μl of 1 M HCl and allowed to incubate at 4 °C. Samples were then centrifuged for 2 min at 2000 rpm and the supernatants were transferred to a fresh tube. Solid phase extraction C-18 cartridges were prepared by washing with 150 μl of methanol followed by 250 μl of double distilled water. Samples were applied on the column and allowed to flow through by centrifugation at 2000 rpm. The column for each sample was rinsed with 250 μl of double distilled water followed by 250 μl of hexane. After the hexane rinse, each column was allowed to dry for 10 min. CysLTs were eluted with 150 μl of methanol. Finally, the samples were dried and 200 μl of assay buffer were added.

#### Assay of cysteinyl leukotriene

Cysteinyl leukotrienes were measured using an ELISA kit, a product of Invitrogen, USA. This ELISA kit is a competitive immunoassay for the quantitative determination of CysLTs in plasma. The kit measures three of the major metabolites of LTA4, LTC4, LTD4 and LTE4.

#### Assay of 8-isoprostane (8-iso-PGF2α)

The levels of 8-isoprostane were measured using a non-radioactive, safe ELISA kit, a product of MyBioSource. This assay employs the quantitative sandwich enzyme immunoassay technique. The minimum detectable concentration of human 8-isoprostane was typically less than 19.5 pg/ml.

#### Statistical analysis

SPSS computer software was used for statistical analyses. Results were expressed as the mean ± SD and all statistical comparisons were made by independent *t*-tests with *p* ≤ 0.05 considered as significant. Receiver Operating Characteristic (ROC) analysis was performed as a comprehensive way to measure the accuracy of the assessed biomarkers. The area under the curve (AUC) provided a useful metric to compare different biomarkers. An AUC value close to 1 indicated an excellent diagnostic and predictive marker; however, a curve that was close to the diagonal (AUC = 0.5) had no diagnostic utility. An AUC close to 1 is always accompanied by satisfactory values of specificity and sensitivity of the biomarker. Moreover, the predictiveness diagrams of the measured parameters were drawn, in which the x-axis represents percentile rank of the biomarker, the y-axis represents the probability of identifying the disease, and the horizontal line is the prevalence of the disease as determined by the Biostat 16 computer program.

## Results

**Fig. 1:**
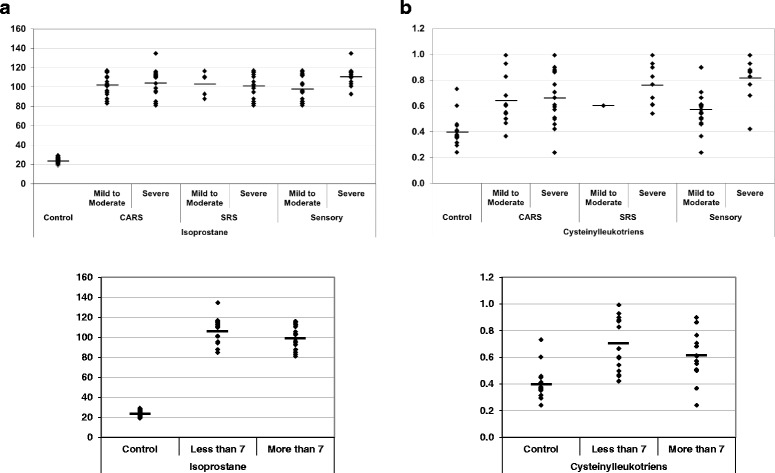
**a** 8-isoprostaneand (**b**) CysLTs levels in autistic and control age groups. The mean value for each group is designated by a line

**Fig. 2:**
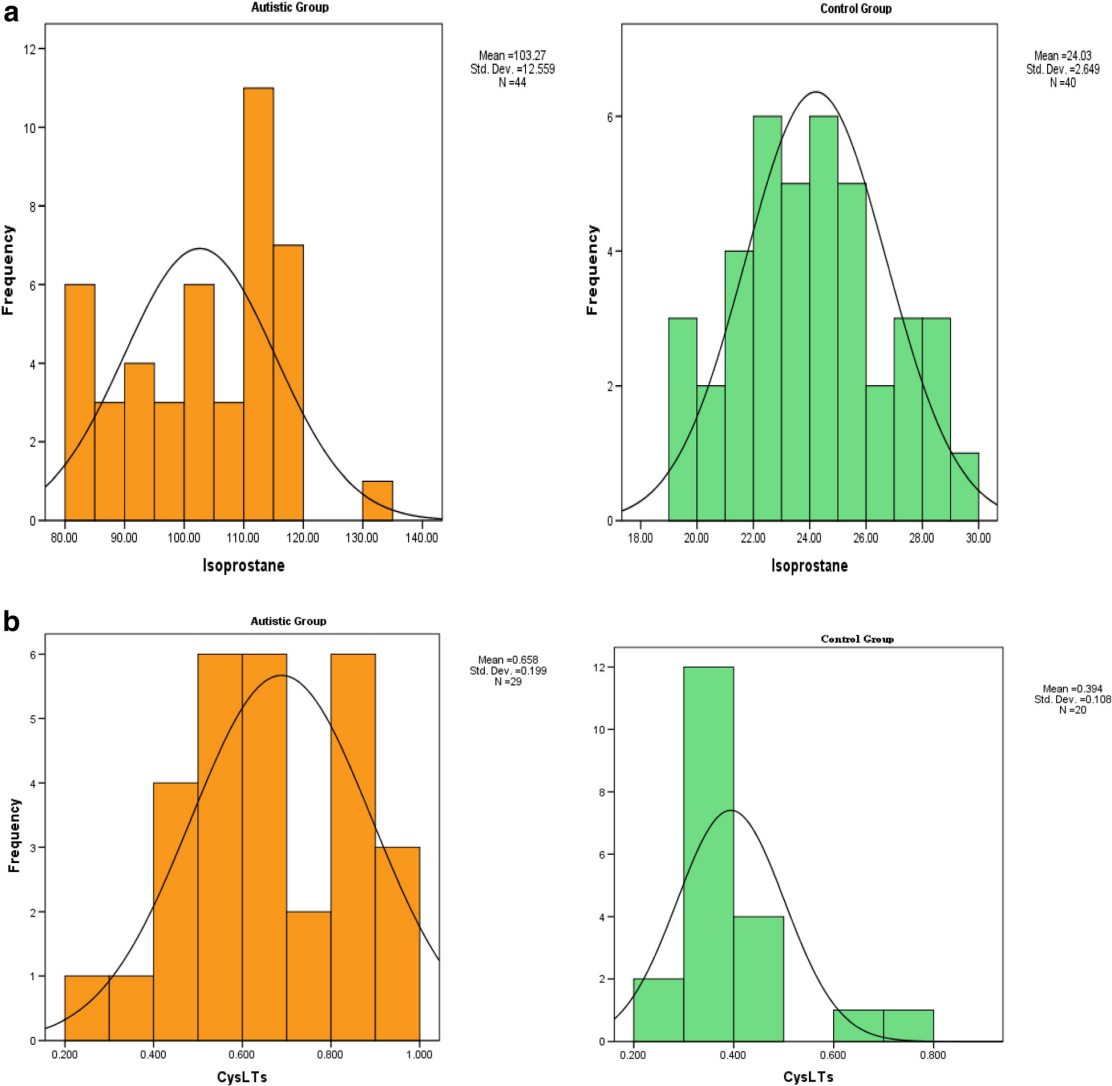
Normal distribution for control and autistic groups for (**a**) 8-isoprostane (pg/ml) and (**b**) CysLTs (ng/ml)

Levels of 8-isoprostane and CysLTs were compared between autism patients with different degrees of autism severity (mild to moderate or severe) and normally developed controls. Autism patients were classified according to their recorded CARS, SRS and SSP scores with age (i.e., less and more than 7 years) (Table 1). Data are presented as the mean ± SD of a maximum number of 44 autism patients compared to 40 controls. The significant differences between both groups and subgroups of patients with autism were presented in Table 1 and Fig. 1. It was observed that the measured parameters differed significantly between autism patients and controls, while there were no significant differences between subgroups of autistic patients with respect to cognitive and social impairments (mild to moderate and severe). Autistic patients with sensory dysfunction showed strong significance levels between mild to moderate and severe subgroups for both parameters. The levels of 8-isoprostane were significantly correlated with age while CysLTs levels did not show any significant differences. Figure 2 demonstrates a normal distribution for the two studied parameters. Moreover, the figure represents the highest and lowest level of 8-isoprostane andCysLTs both for autistic patients and controls. From the normal distribution (Fig. 2a & b) of 8-isoprostane, it can be easily observed that while the highest recorded value in the control group was 30 pg/ml, the lowest value in the autistic group was 80 pg/ml. For CysLTs, 18 out of 30 control participants exhibited concentration levels less than 0.6 ng/ml; however, 17 out of 29 autistic patients demonstrated CysLTs values higher than 0.6 ng/ml.

**Table 1:** Levels of 8-isoprostane and CysLTs in autistic patients compared to control participants

Parameters	Group	N	Mean ± S.D.	*P* value
8-Isoprostane (pg/ml)	Control	40	24.03 ± 2.65	0.001^*^
	Autistic Patients	44	103.27 ± 12.56	
	Autism (severe in CARS)	21	102.09 ± 11.11	0.556^**^
	Autism (mild to moderate in CARS)	23	104.35 ± 13.91	
	Autism (severe in SRS)	5	103.35 ± 12.47	0.741^**^
	Autism (mild to moderate in SRS)	16	101.19 ± 12.60	
	Autism (mild to moderate in sensory)	24	97.66 ± 12.65	0.001^**^
	Autism (severe in sensory)	17	111.17 ± 8.67	
	Age (less than 7)	23	107.08 ± 12.19	0.034^**^
	Age (more than 7)	21	99.10 ± 11.86	
Cysteinyl leukotrienes (ng/ml)	Control	20	0.394 ± 0.108	0.001^*^
	Autistic Patients	29	0.658 ± 0.199	
	Autism (mild to moderate in CARS)	11	0.642 ± 0.197	0.738^**^
	Autism (severe in CARS)	18	0.668 ± 0.205	
	Autism (mild to moderate in SRS)	1	0.602 ± 0.000	0.382^**^
	Autism (severe in SRS)	9	0.759 ± 0.161	
	Autism (mild to moderate in sensory)	19	0.573 ± 0.156	0.001^**^
	Autism (severe in sensory)	10	0.821 ± 0.170	
	Age (less than 7)	15	0.708 ± 0.210	0.164^**^
	Age (more than 7)	14	0.605 ± 0.177	

^*^ *P* value between control and autistic groups

^**^ *P* value between mild to moderate and severe in CARS, SRS, SSP and age

Sensitivity and specificity are the basic measures of the accuracy of a diagnostic test. These parameters describe the ability of a test to correctly diagnose disease when disease is actually present and to correctly rule out disease when disease is truly absent. ROC show that AUC for 8-isoprostane was one, while that for CysLTs was slightly less than one. By using a statistical analysis program (SPSS) which includes the Pearson correlation test, a correlation was made between all parameters; the results showed that there was a correlation significance difference between the parameters shown in Table 2. For autistic individuals, the relationship between the levels of 8-isoprostane, CysLTs and severity of autism that was measured by the CARS, SRS, and SSP scores were also evaluated. There was a positive correlation between 8-isoprostane and CysLTs (*R* = 0.686, *p* < 0.001) and there were negative correlations between 8-isoprostane, CysLTs, and SSP scores (*R* = −0.517, *p* < 0.05 and *R* = −0.615, *p* < 0.001). Additionally, age had a significant negative correlation with 8-isoprostane plasma levels (*R* = −0.376, *p* < 0.012) and had a significant positive correlation with SSP scores (*R* = 0.296, *p* < 0.043).

**Table 2:** Pearson’s correlations between the different studied variables

Parameters	R (Pearson Correlation)	Sig.
SSP ~ Age	0.296^b^	0.043
SSP ~ 8-isoprostane (pg/ml)	−0.517^a^	0.001
SSP ~ CysLTs (ng/ml)	−0.615^a^	0.001
Age ~ 8-isoprostane (pg/ml)	−0.376^b^	0.012
8-isoprostane (pg/ml) ~ CysLTs(ng/ml)	0.686^a^	0.001

^a^ Correlation is significant at the 0.001 level

^b^ Correlation is significant at the 0.05 level

## Discussion

It is well known that oxidative stress plays a critical role in many neurodevelopmental disorders [47, 48]. 8-isoprostane is the most characterized compound that provided a reliable index of unique bioactive products of lipid peroxidation and oxidative stress in a variety of clinical settings.

In the present study, significant increase in the level of 8-isoprostane was recorded in autistic patients compared to controls. This can demonstrate the role of inflammation as etiological mechanism in autism as it is consistent with previous studies that show high 8-isoprostane level in plasma, urine or breath from patients suffering from inflammation [49–52]. The normal range of 8-isoprostane in the blood of humans is between 20 and 80 pg/ml, and based on the data obtained herein, 8-isoprostane levels in autistic children are much higher than the upper limit of the normal range [53]. In the present study, although a four-fold increase in 8-isoprostane was detected in autistic children compared to controls, a relationship between 8-isoprostane and social or cognitive impairments was completely absent. However, a significant correlation between 8-isoprostane and sensory dysfunction was clearly observed. The significant difference in the 8-isoprostane level between the two studied age groups (^<^7 or >7) can help to suggest that oxidative stress is an early event that may play an important role in the pathogenesis of autism. The observed increase in 8-isoprostane are in good agreement with many previous studies that collectively demonstrate the usefulness of this marker for diagnosing autism [15, 54, 55]. In a previous study conducted by Ming et al., a significant increase of l urinary excretion of 8-isoprostane was recorded in children with autism compared to age-matched controls [15]. Plasma levels of 8-isoprostane have also been correlated with anti-neural antibodies as a measure of autoimmunity as a pathological mechanism in autism [54, 56].

Moreover, the significantly high level of 8-isoprostane reported in the present study may be related to the brain hypoperfusion that has been reported in autistic children compared to controls. Yuemang et al. observed a correlation between 8-isoprostane and abnormal blood flow in autism because of the role of 8-isoprostane in platelet aggregation and vaso-constriction [57]. Moreover, multiple neuroimaging studies have noted the relationship between oxidative stress and vascular homeostasis in the pathogenesis of autism, including the possible influence of 8-isoprostane on tissue perfusion [58, 59].

Glutamate is the main excitatory neurotransmitter in the CNS; it plays an important role in the brain and can cause damage in response to neurological dysfunction. A strong correlation between 8-isoprostane and glutamate excitotoxicity as a phenotype repeatedly recorded in autism has been reported in several studies [47, 60–62].

There is increasing evidence that CysLTs may play a role in CNS activity. In the present study, although the levels of CysLTs were significantly higher in autistic children compared to healthy controls, there was no association with age, CARS, or SRS. Contrarily, CysLTs levels were significantly associated with the severity of sensory dysfunction.

The deleterious effects of CysLTs usually occur through the induction of superoxide radicals as a marker of oxidative stress [63]. While CysLTs do not directly stimulate sensory nerves, they induce the release of increased amounts of neuropeptides from tachykinergic nerves under the influence of action potentials [64, 65]. This suggests that CysLTs may potentiate neural phenomena such as neurogenic inflammation, and excitotoxicity [66, 67]. In different neurological diseases, CysLT1 receptor mRNA has been found to be up-regulated in neurons, macrophages and proliferated astrocytes, suggesting a possible regulatory role in the mediation of different phases of neuro-inflammation [68, 69]. These compounds have been associated with brain edema formation and disruption of the BBB [70]. The obtained alteration in 8-isoprostane and CysLTs reported in the present study, can find support in the record of Das [71] showing that changes in the metabolism of AA and other PUFAs result in excess production of proinflammatory cytokines and inflammatory lipid mediators and less production of anti-inflammatory cytokines and bioactive lipids that ultimately induce the development of autism.

The significant positive correlations discovered herein between 8-isoprostane and CysLTs (Table 2) confirmed an association between impaired phospholipid metabolism, inflammation, nutritional status and oxidative stress in the etiopathology of autism [68–72]. The discovered negative correlation between 8-isoprostane as a marker of oxidative stress and age may explain the remarkable improvement of autistic patients near adulthood. This suggestion has been supported in two previous studies that showed an improvement of phenotypes in a majority of autistic patients during the transition to adulthood [73, 74]. Fletcher-Watson et al. [75] concluded that adults with autism were faster and more accurate at detecting eye gaze than controls. Moreover, restricted repetitive behavior was less frequent and less severe among older individuals than younger individuals, suggesting that autism may somewhat diminishes with age.

**Fig. 3:**
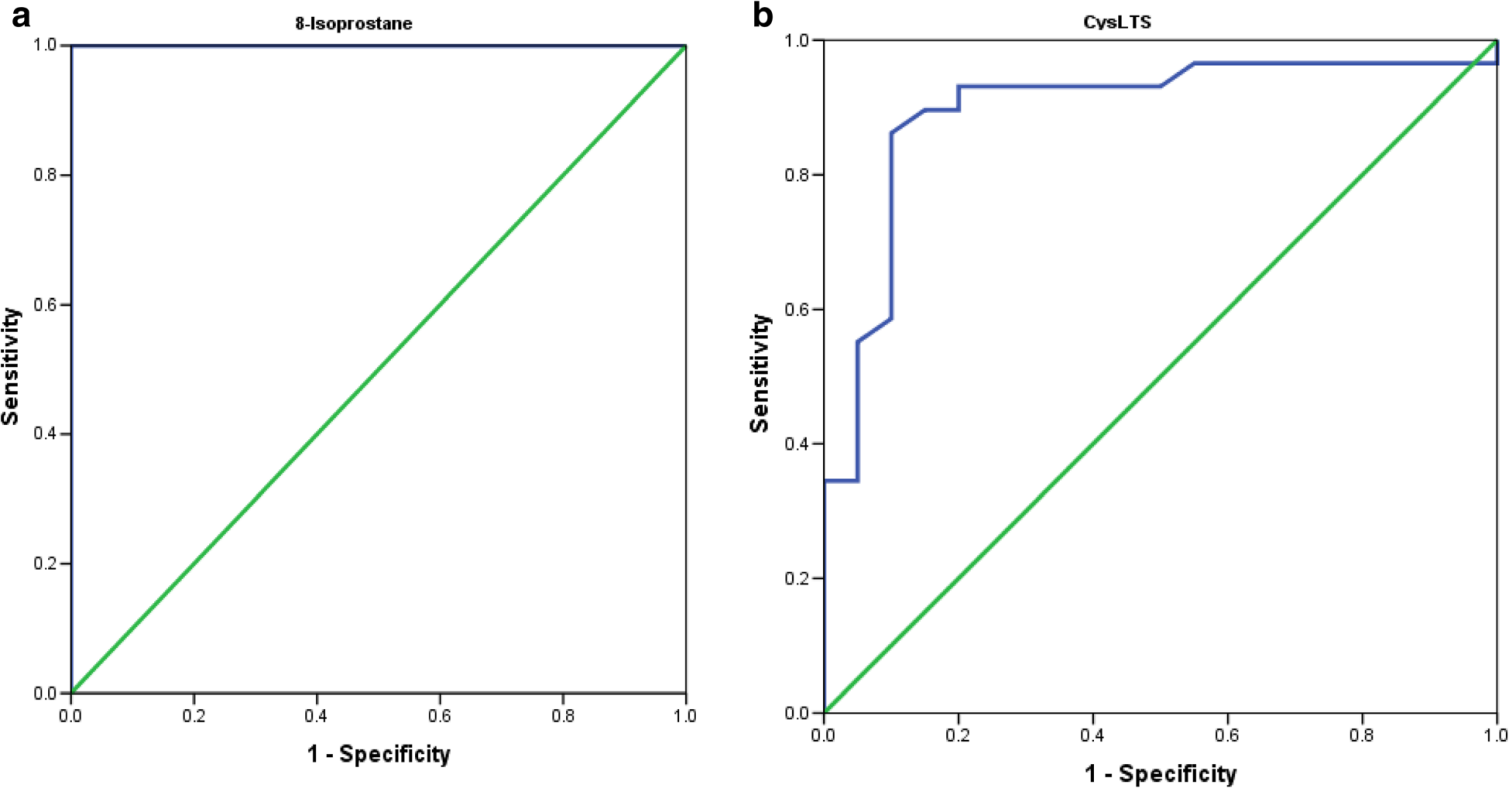
ROC curves for 8-isoprostane and CysLTs

**Fig. 4:**
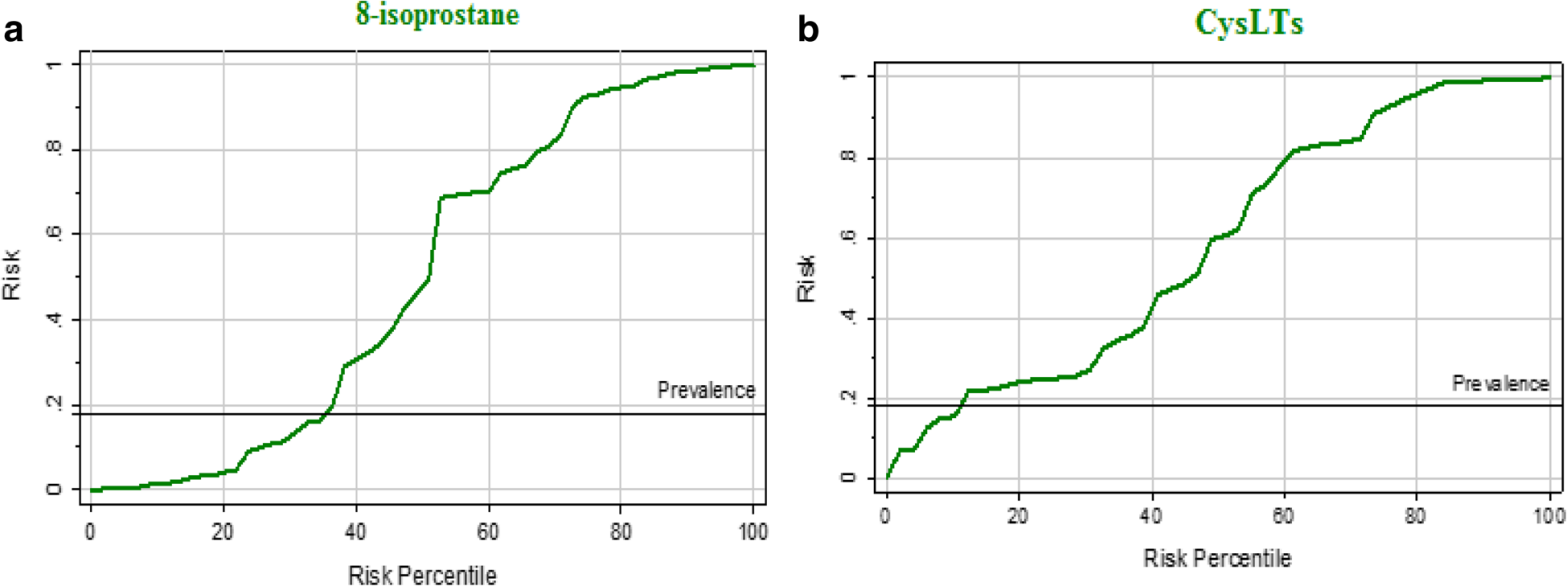
The predictiveness curves as an assessment of the performance of 8-isoprostane and CysLTs in autism risk prediction in the Saudi population. The two measured parameters showed adequate predictive power

Although there was no correlation between CARS, SRS or either of the oxidative stress markers in the present study, previous studies have shown that oxidative stress causes cognitive decline and memory loss [76]. The effectiveness of using both of the measured parameters for the correct diagnosis of autism can be easily supported through ROC analysis (Table 3 and Fig. 3 a and b). Both biomarkers exhibited high AUC values together with satisfactory specificity and sensitivity. In fact, a key issue in performing an intervention trial with antioxidants is establishing whether the treatment decreases oxidative stress in study subjects and at what dose. Measurement of the level of lipid peroxidation, which is reflected by 8-isoprostane concentrations in biological fluids, may help to identify patients who are most likely to benefit from antioxidant treatments [77, 78]. This can be supported by the illustrated predictiveness curves (Fig. 4 a and b). While both of the measured oxidative stress biomarkers demonstrate perfect predictiveness curves herein, 8-isoprostane shows more predictive value than CysLT1 and thus can be used to follow-up future suggested antioxidant intervention strategies.

**Table 3:** Values of area under the ROC curve (AUC), sensitivity and specificity for the optimal cut-off point for mild to moderate CARS, SRS, SSP and age

Parameters	Scales	AUC	Cutoff value	Sensitivity %	Specificity%
8-Isoprostane (pg/ml)	CARS	1.000	56.165	100.0	100.0
	SRS	1.000	58.465	100.0	100.0
	SSP	1.000	55.049	100.0	100.0
	Age (Less than 7 years)	1.000	56.981	100.0	100.0
	Age (More than 7 years)	1.000	55.049	100.0	100.0
CysLTs (ng/ml)	CARS	0.900	0.463	90.9	90.0
	SRS	0.925	0.530	100.0	90.0
	SSP	0.854	0.453	89.5	85.0
	Age (Less than 7 years)	0.943	0.416	100.0	80.0
	Age (More than 7 years)	0.845	0.479	85.7	90.0

## Conclusions

In summary, the data presented herein indicate that 8-isoprostane and CysLTs are reliable biomarker for oxidative stress that present in autism, and can be easily measured from a blood sample. 8-isoprostane and CysLTs, known as lipid mediators that are excreted by activated glial cells under stimulation of ROS. Many studies of autism revealed that those lipid mediators arising from neuro-inflammation is consistent with all biochemical, pathophysiological data that done on autism patients and can easily related to the nutritional factors [72, 79].

## Abbreviations

AA, arachidonic acid; CARS, Childhood autism rating scale; COX-2, cyclooxygenase-2; cPLA2, cytosolic phospholipase A2; CysLTs, cysteinyl leukotrienes; GSH, glutathione; IFNγ, interferon gamma; IL-1, interleukin-1; IL-6, interleukin-6; PGE2, prostaglandin E2; PUFAs, polyunsaturated fatty acids; ROC-curve, receiver operating characteristics curve; ROS, reactive oxygen species; SRS, social responsiveness scale; SSP, short sensory profile; TNF-α, tumor necrosis factor alpha
